# Anakinra Reduces Epileptogenesis, Provides Neuroprotection, and Attenuates Behavioral Impairments in Rats in the Lithium–Pilocarpine Model of Epilepsy

**DOI:** 10.3390/ph13110340

**Published:** 2020-10-25

**Authors:** Alexandra V. Dyomina, Olga E. Zubareva, Ilya V. Smolensky, Dmitry S. Vasilev, Maria V. Zakharova, Anna A. Kovalenko, Alexander P. Schwarz, Alexander M. Ischenko, Aleksey V. Zaitsev

**Affiliations:** 1 Sechenov Institute of Evolutionary Physiology and Biochemistry of RAS, 44, Toreza Prospekt, 194223 Saint Petersburg, Russia; adyomina513@gmail.com (A.V.D.); zubarevaoe@mail.ru (O.E.Z.); smolensky.ilya@gmail.com (I.V.S.); dvasilyev@bk.ru (D.S.V.) zaharova-masha@yandex.ru (M.V.Z.); kovalenko_0911@mail.ru (A.A.K.); aleksandr.pavlovich.schwarz@gmail.com (A.P.S.); 2 Research Institute of Highly Pure Biopreparations, Federal Medical-Biological Agency, 7, Pudozhskaya Street, 197110 Saint Petersburg, Russia; a.m.ischenko@hpb.spb.ru

**Keywords:** temporal lobe epilepsy, anakinra, lithium–pilocarpine model, behavior, epileptogenesis, hippocampus, spontaneous recurrent seizures, gliosis, *Il1b*, *Tnfa*, *Gfap*, *Itpr2*, *Slc1a2*, *mRNA*

## Abstract

Temporal lobe epilepsy is a widespread chronic disorder that manifests as spontaneous seizures and is often characterized by refractoriness to drug treatment. Temporal lobe epilepsy can be caused by a primary brain injury; therefore, the prevention of epileptogenesis after a primary event is considered one of the best treatment options. However, a preventive treatment for epilepsy still does not exist. Neuroinflammation is directly involved in epileptogenesis and neurodegeneration, leading to the epileptic condition and cognitive decline. In the present study, we aimed to clarify the effect of treatment with a recombinant form of the Interleukin-1 receptor antagonist (anakinra) on epileptogenesis and behavioral impairments in rats using the lithium–pilocarpine model. We found that anakinra administration during the latent phase of the model significantly suppressed the duration and frequency of spontaneous recurrent seizures in the chronic phase. Moreover, anakinra administration prevented some behavioral impairments, including motor hyperactivity and disturbances in social interactions, during both the latent and chronic periods. Histological analysis revealed that anakinra administration decreased neuronal loss in the CA1 and CA3 areas of the hippocampus but did not prevent astro- and microgliosis. The treatment increased the expression level of the solute carrier family 1 member 2 gene (*Slc1a2,* encoding excitatory amino acid transporter 2 (EAAT2)) in the hippocampus, potentially leading to a neuroprotective effect. However, the increased gene expression of proinflammatory cytokine genes (Interleukin-1β (*Il1b*) and tumor necrosis factor α (*Tnfa*)) and astroglial marker genes (glial fibrillary acidic protein (*Gfap*) and inositol 1,4,5-trisphosphate receptor type 2 (*Itpr2*)) in experimental rats was not affected by anakinra treatment. Thus, our data demonstrate that the administration of anakinra during epileptogenesis has some beneficial disease-modifying effects.

## 1. Introduction

Epilepsy is a widespread chronic disorder that manifests as spontaneous seizures [1]. The disease can lead to educational, vocational, and action (e.g., driving) limitations, traumas, and increased mortality [2]. Frequently, progressive epilepsy becomes refractory, and nearly 30% of cases do not respond to drug therapy [3]. Moreover, epilepsy may be accompanied by psychiatric comorbidities, such as depression, anxiety, psychosis, cognitive impairment, sleep disorders, and migraine [4], which also decrease quality of life and complicate therapy.

Traumatic factors (infections and injuries), tumors, status epilepticus (SE), and perinatal complications can frequently cause acquired epilepsy [5]. In these cases, seizures usually develop after a latent period. Thus, antiepileptogenic therapy during the latent period is considered the best option to prevent epilepsy [6,7]. However, a preventive treatment for epilepsy still does not exist [8]. The development of rational preventive therapy requires knowledge of the exact mechanisms of epileptogenesis. Several pathogenic mechanisms are involved in epileptogenesis, one of which is neuroinflammation [9,10]. Therefore, anti-inflammatory therapy in susceptible groups could reduce the risk of the development of epilepsy.

There are several potential targets of anti-inflammatory therapy, including inflammatory intracellular and extracellular mediators (cytokines, high-mobility group protein 1 (HMGB1), prostaglandins, NO, and complement system) and their receptors, enzymes (cyclooxygenase-2 (COX-2)), and transcription factors (nuclear factor kappa-light-chain-enhancer of activated B cells (NFкB) and activator protein 1 (AP-1)) [11]. In the brain, extracellular mediators not only interact with immune and vessel cells but also change neuronal excitation and synaptic activity. Thus, they may contribute to epileptogenesis and associated pathological processes, including neuronal damage and excitatory/inhibitory disbalance [12].

The Interleukin-1 (IL-1) family, which includes 11 cytokines, plays a central role in neuroinflammation and neuronal activity modulation. IL-1 alpha (IL-1α) and beta (IL-1β) are the most studied members of this family, and they possess a robust proinflammatory effect, which is implemented by the membrane IL-1 receptor, type I (IL-1R1) [13]. IL-1 receptor antagonist (IL-1Ra) regulates IL-1α and IL-1β proinflammatory activity by competing with them for IL-1R1 binding sites [14].

The expression of IL-1β genes is enhanced in patients with epilepsy, and its increase is associated with the severity of seizures in animal models of epilepsy [15,16,17]. In seizure models, exogenously applied IL-1β prolongs seizures in an IL-1R1-mediated manner [18]. It is hypothesized that the IL-1β/IL-1R1 axis provides the recurrent character of chronic seizures and promotes seizure onset after brain injury [19]. IL-1Ra administration could be a potential approach for the preventive treatment of epilepsy. A previous study by Noe et al. [20] showed that a combination of two anti-inflammatory drugs, human recombinant IL-1Ra (anakinra) and VX-765 (a specific nonpeptide inhibitor of IL-1β cleavage and release), significantly decreased both IL-1β expression in astrocytes and cell loss in rat forebrains. However, this treatment did not prevent epilepsy development in either pilocarpine or electrically induced SE epilepsy models [20]. In another study, IL-1Ra treatment improved cognitive function and decreased seizure susceptibility after pediatric brain injury in mice [21].

The present study aims to clarify the effect of anakinra treatment on epileptogenesis and comorbid behavioral impairments in rats using the lithium–pilocarpine model. This model was chosen because it is one of the most effective for reproducing epileptogenesis [22]. Anakinra was administered for 10 days following pilocarpine-induced SE. To estimate the efficacy of the therapy, we assessed the frequency and duration of spontaneous recurrent seizures (SRS), analyzed the effect of treatment on astrogliosis and neuronal loss, and evaluated changes in the gene expression of Il-1β, tumor necrosis factor alpha (TNFα), and glial fibrillary acidic protein (GFAP). We also investigated if anakinra treatment ameliorates disturbances in locomotor and exploratory behavior, cognitive functions, and social interactions in experimental animals.

## 2. Results

### 2.1. Survival and Body Weight Dynamics after SE

**Figure 1:**
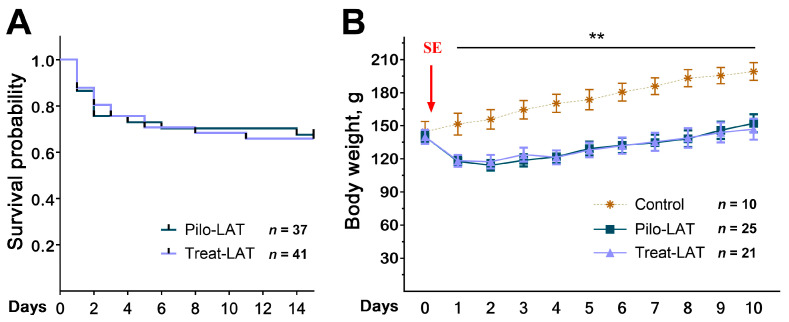
Survival and body weight dynamics following pilocarpine-induced status epilepticus (SE). (**A**) The Kaplan–Meier survival curves show no effect of anakinra treatment on rat survival. (**B**) Bodyweight dynamics. The red arrow shows the day when SE was induced. Repeated measures ANOVA with Tukey’s honest significant difference (HSD) post hoc test. F_(20,58)_ = 2.33, *p* < 0.007. ** *p* < 0.01 between control and both post-SE groups. Pilo-LAT: rats with pilocarpine-induced SE observed during the latent phase; Treat-LAT: post-SE rats treated with anakinra observed during the latent phase.

Anakinra treatment did not change the rat survival in the 14 days following SE (Figure 1A). We found the same mortality rate in both rat groups; most rats died in the first three days after SE. The treatment did not affect the dynamics of rat weight after SE (Figure 1B). In the control group, the animals’ weight increased during the entire observation period, while in the post-SE groups, the weight decreased during the first days after SE regardless of treatment. Weight regain began only 3–4 days after SE.

### 2.2. Anakinra Treatment Reduced the Frequency and Duration of SRS

**Figure 2:**
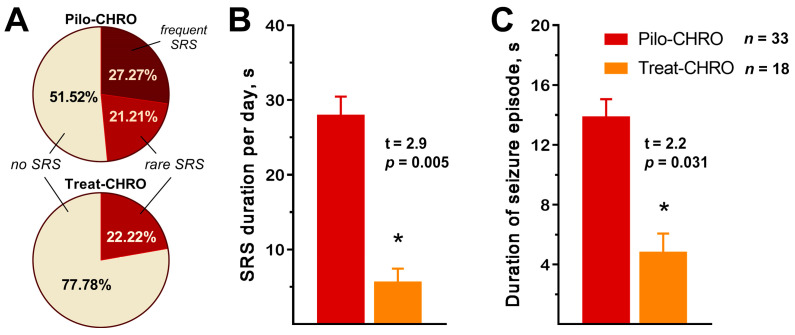
Anakinra treatment reduced the frequency and duration of spontaneous recurrent seizures (SRSs) in the chronic phase of the lithium–pilocarpine model. (**A**) Percentage of rats with SRS. Rare SRS: 1–2 seizure episodes; frequent SRS: 3 or more episodes. (**B**) The total SRS duration per 24 h was shorter in the treatment group. (**C**) The average time of each SRS episode was shorter in the treated animals. Pilo-CHRO: rats with pilocarpine-induced SE observed during the chronic phase; Treat-CHRO: post-SE rats treated with anakinra observed during the chronic phase. * *p* < 0.05 in *t*-test.

SRSs were observed in ~50% of Pilo-CHRO rats compared to only ~20% of treated animals (Figure 2A). The number of SRSs per day was also lower in the treated group (χ^2^ = 6.24, *p* = 0.04). Anakinra treatment reduced the total seizure duration per day by almost five times (Figure 2B) and the average duration of each seizure was three times shorter in the treated group (Figure 2C). Therefore, anakinra therapy during the latent phase has a beneficial antiepileptogenic effect.

### 2.3. Anakinra Treatment Reduced Neuronal Loss but Did Not Prevent Gliosis

**Figure 3:**
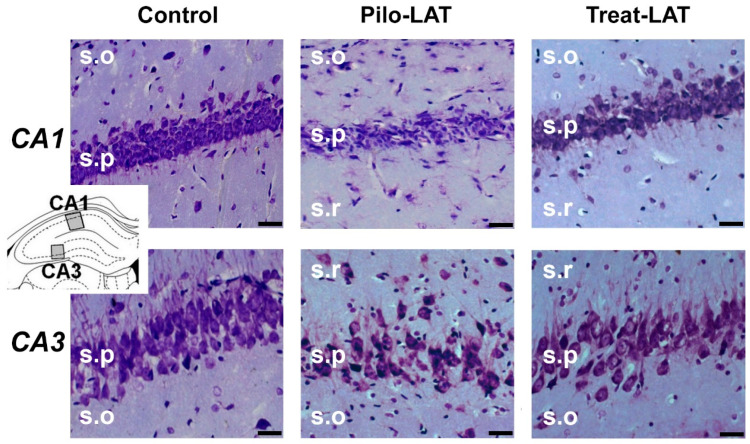
Neurodegeneration and gliosis after SE (latent phase). Nissl staining showing neurodegeneration in the *str. pyramidale* (s.p.) of CA1 and CA3 areas. s.o.: *stratum oriens*; s.r.: *stratum radiatum*. The insert shows the schema of the hippocampus with the sites for morphological analysis. Pilo-LAT: rats administered with pilocarpine; Treat-LAT: rats administered with pilocarpine and then treated with anakinra. The scale bar is 30 µm.

Next, we examined if anakinra administration had a neuroprotective effect. Nissl staining revealed substantial neurodegeneration and gliosis in the pyramidal layers of the CA1 and CA3 areas of the hippocampus following pilocarpine-induced SE (Figure 3, middle column). Anakinra treatment partially precluded neuronal loss; however, the increase in the number of glial cells was not prevented (Figure 3, right column).

**Figure 4:**
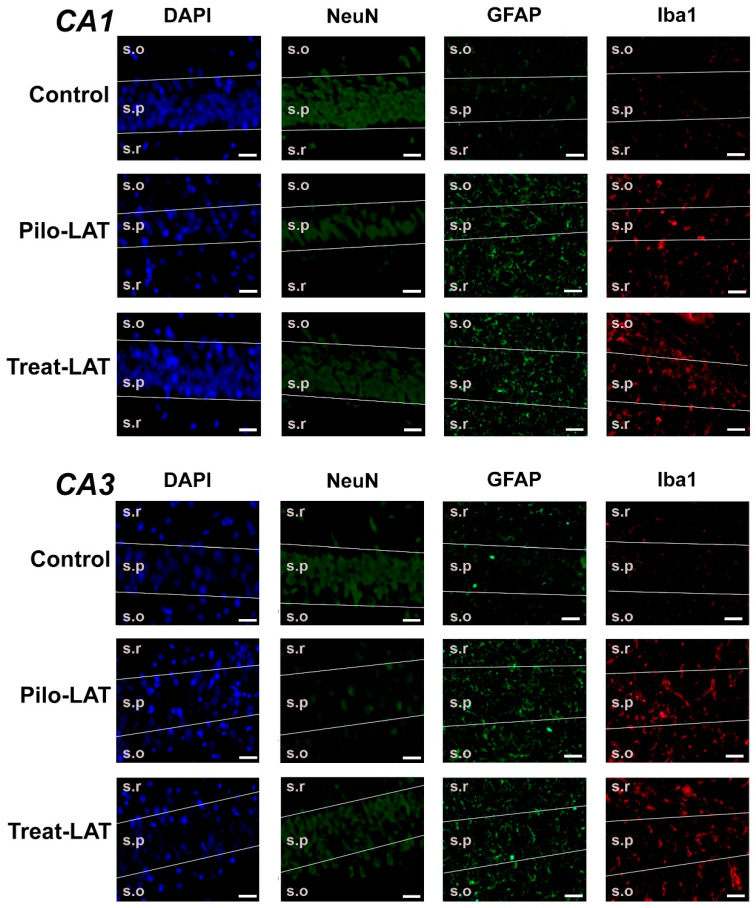
The effects of anakinra treatment on the distribution of neuronal (NeuN) and glial (glial fibrillary acidic protein (GFAP) and Iba1) marker proteins in the CA1 and CA3 hippocampus of rats following pilocarpine-induced SE. Cell nuclei were stained by DAPI (blue). The neuronal marker NeuN and astrocyte marker GFAP were visualized with phycoerythrin-conjugated (FITC) secondary antibodies (green). The microglia marker protein Iba was visualized with FITC secondary antibodies. Pilo-LAT: rats administered with pilocarpine; Treat-LAT: rats administered with pilocarpine and then treated with anakinra. s.p.: *str. pyramidale* of CA1, CA3 areas; s.o.: *stratum oriens*, s.r.: *stratum radiatum*. Scale bar is 30 µm.

**Figure 5:**
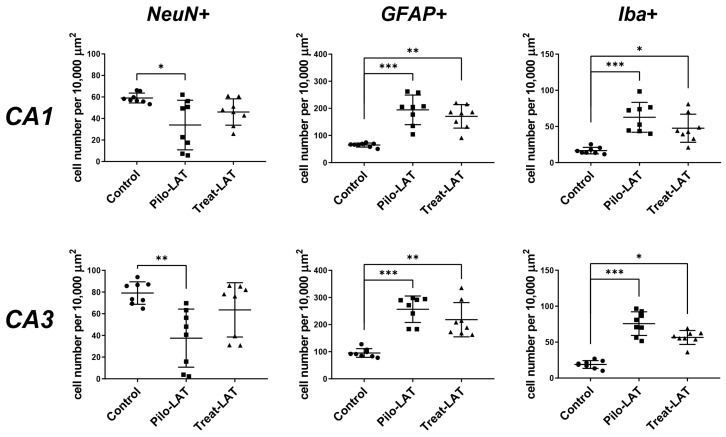
Effects of anakinra treatment on neuronal loss and gliosis. Diagrams show the number of NeuN-positive neurons, GFAP-positive astrocytes, and Iba-positive microglial cells in the CA1 and CA3 areas of the hippocampus. Each dot represents results from one animal; horizontal lines show the means and standard deviations. Group comparisons were made with the Kruskal–Wallis test and followed with Dunn’s post hoc tests. NeuN-positive cells: CA1 (F_(2,23)_ = 7.8, *p* = 0.02), CA3 (F_(2,23)_ = 9.8, *p* < 0.01), GFAP-positive astrocytes: CA1 (F_(2,23)_ = 15.7, *p* < 0.001), CA3 (F_(2,23)_ = 16.1, *p* < 0.001), Iba-positive microglial cells: CA1 (F_(2,23)_ = 16.1, *p* < 0.001), CA3 (F_(2,23)_ = 17.6, *p* < 0.001). Pilo-LAT: rats administered with pilocarpine; Treat-LAT: rats administered with pilocarpine and then treated with anakinra. * *p* < 0.05; ** *p* < 0.01; *** *p* < 0.001.

For the quantitative analysis, we performed an immunofluorescent assay using neuronal marker NeuN, astrocyte marker GFAP, and microglia cell marker Iba1. The number of NeuN-positive neurons was significantly decreased in the CA1 and CA3 areas following SE. Consistent with previous reports [23], the average number of GFAP-positive astrocytes was almost three times higher in the Pilo-LAT group than in the control group. The number of Iba1-positive microglial cells in the CA1 and CA3 of the Pilo-LAT group was about four times higher than in the control group (Figure 4 and Figure 5).

Anakinra treatment reduced neuronal loss since no significant differences in NeuN-positive cells in the CA1 and CA3 areas were found between the control and Treat-LAT groups (Figure 4 and Figure 5). However, the number of GFAP-positive astrocyte cells and Iba1-positive microglial cells in the Treat-LAT group was higher than in the control group and did not differ from the Pilo-LAT group. We concluded that anakinra treatment has a significant neuroprotective effect, but it failed to prevent gliosis in rats subjected to pilocarpine-induced SE.

### 2.4. Changes in Gene Expression of Cytokines and Astroglia Activation Markers

**Figure 6:**
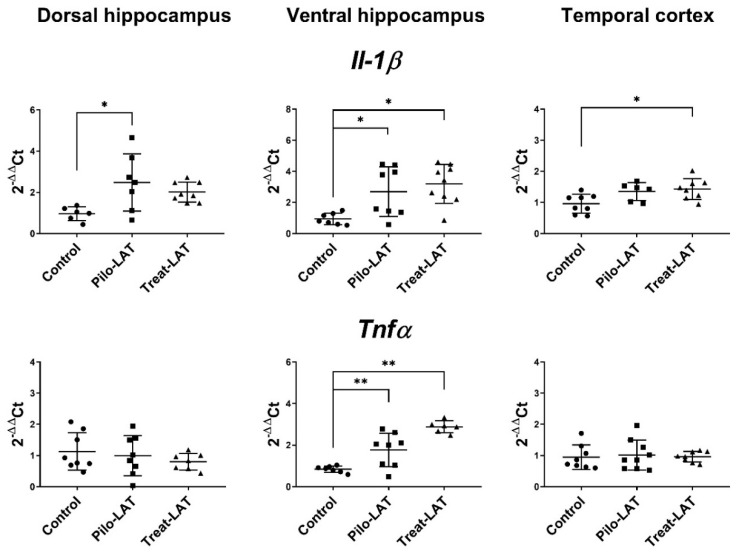
The relative expression of proinflammatory cytokine genes (*Il1b* and *Tnfα*) in the dorsal hippocampus (DH), ventral hippocampus (VH), and temporal cortex (TC) of rats 7 days after pilocarpine-induced SE without (Pilo-LAT) and with (Treat-LAT) anakinra treatment. Each dot represents one animal; horizontal lines show the means and standard deviations. * *p* < 0.05; ** *p* < 0.01 in ANOVA with Tukey’s post hoc test. *Il1b*: DH: F_(2,18)_ = 5, *p* = 0.02; VH: F_(2,21)_ = 7.1, *p* = 0.004; TC: F_(2,19)_ = 5, *p* = 0.02. *Tnfa*: VH: F_(2,18)_ = 23.5, *p* < 0.001.

Gene expression of proinflammatory cytokine genes (*Il1b* and *Tnfa*) and astroglial marker genes (*Gfap*, *Slc1a2*, and *Itpr2*) was evaluated in the dorsal hippocampus (DH), ventral hippocampus (VH), and temporal cortex (TC) seven days after pilocarpine-induced SE. At this time point, anakinra treatment was ongoing. For the experimental animals, *Il1b* mRNA expression was upregulated in all examined brain areas, while *Tnfa* mRNA level was increased only in the VH (Figure 6). The anakinra treatment did not prevent the increased expression of proinflammatory cytokine genes.

**Figure 7:**
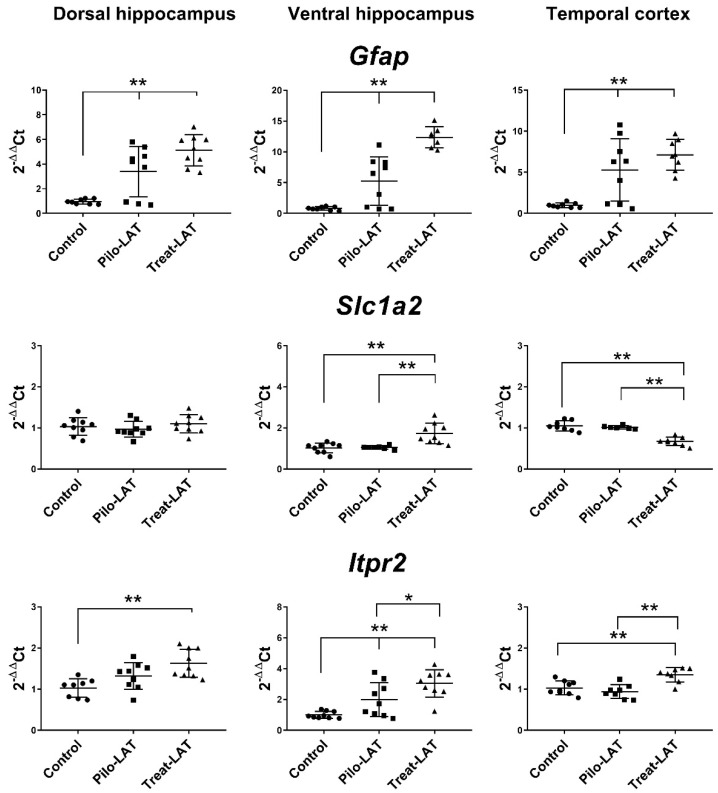
The relative expression of astroglial marker genes (*Gfap*, *Slc1a2*, *Itpr2*) in the dorsal hippocampus (DH), ventral hippocampus (VH), and temporal cortex (TC) of rats seven days after pilocarpine-induced seizures (Pilo-LAT) and after SE following anakinra treatment (Treat-LAT). Each dot represents one animal; horizontal lines show the means and standard deviations. * *p* < 0.05; ** *p* < 0.01 in ANOVA with Tukey’s post hoc test. *Gfap*: DH: F_(2,23)_ = 18.3, *p* < 0.001; VH: F_(2,21)_ = 37.4, *p* < 0.001; TC: F_(2,22)_ = 12.5, *p* < 0.001. *Slc1a2*: VH: F_(2,23)_ = 12.5 *p* < 0.001; TC: F_(2,19)_ = 34.7, *p* < 0.001. *Itpr2*: DH: F_(2,23)_ = 8.3, *p* = 0.002; VH: F_(2,24)_ = 13.8, *p* < 0.001; TC: F_(2,22)_ = 12.9, *p* < 0.001.

Next, we analyzed the gene expression of the astroglia markers *Gfap*, *Slc1a2*, and *Itpr2* (Figure 7). An increased level of *Gfap* mRNA was revealed in all tested brain areas. Anakinra treatment enhanced these changes in both hippocampal regions but did not affect the TC. *Slc1a2* and *Itpr2* gene expressions remained at the basal level in untreated animals after pilocarpine-induced SE. Anakinra treatment upregulated *Itpr2* gene expression in all examined brain areas and *Slc1a2* expression in the VH but downregulated *Slc1a2* gene expression in the TC (Figure 7).

### 2.5. Behavioral Alterations

The behavior of post-SE animals was significantly disturbed in most tests during the latent and chronic periods [24]. Therefore, we investigated whether anakinra treatment improved the results for the animals in both periods.

#### 2.5.1. Locomotor Activity in the Open-Field Test (OFT)

**Figure 8:**
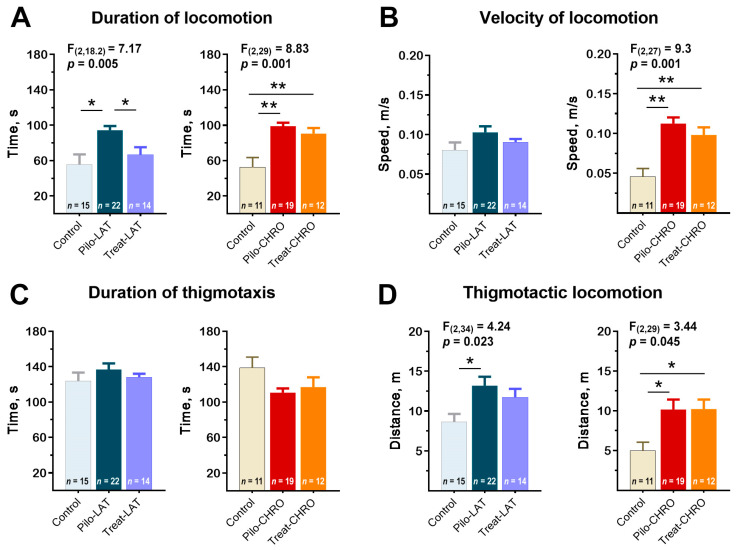
Effects of anakinra treatment on locomotor activity in the open-field arena. One-way ANOVA with Tukey’s post hoc test was used for statistical analysis. Significant differences between groups are shown in the diagrams. (**A**) Duration of locomotion in the open-field test (OFT). (**B**) The average speed of locomotion. (**C**) The average time of locomotion in the thigmotactic area. (**D**) Distance traveled in the thigmotactic area. Pilo-LAT and Pilo-CHRO: rats with pilocarpine-induced SE studied during the latent phase and chronic phase of the model accordingly; Treat-LAT and Treat-CHRO: post-SE rats treated with anakinra and studied during the latent phase and chronic phase of the model. * *p* < 0.05, ** *p* < 0.01.

Pilo rats were more active in the open-field arena; they spent more time moving around the arena than the control animals (Figure 8A). Pilo-CHRO rats moved faster than rats from the control group (Figure 8B). The difference between groups was mostly due to the higher activity of Pilo rats in the thigmotactic area. The thigmotactic locomotion time was the same for all groups, but the thigmotactic distance was increased in the post-SE groups compared to the control group (Figure 8C,D).

Anakinra treatment effectively prevented rat hyperactivity in the latent phase. However, it did not affect the properties of locomotor activity in the chronic period (Figure 8).

#### 2.5.2. Exploratory Behavior in the OFT

**Figure 9:**
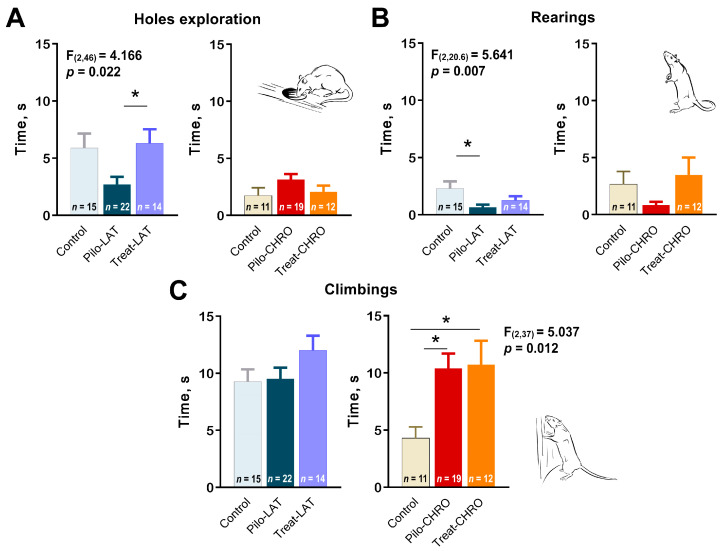
Effects of anakinra treatment on characteristics of exploratory activity in post-SE rats. One-way ANOVA with Tukey’s post hoc test was used for statistical analysis. Significant differences between groups are shown in the diagrams. Diagrams show the average time spent (**A**) exploring holes, (**B**) rearing, and (**C**) climbing for the different groups of rats. Pilo-LAT and Pilo-CHRO: rats with pilocarpine-induced SE studied during the latent phase and chronic phase of the model accordingly; Treat-LAT and Treat-CHRO: post-SE rats treated with anakinra and studied during the latent phase and chronic phase of the model. * *p* < 0.05, Tukey’s post hoc test.

Next, we analyzed exploratory behavior in the OFT. We observed a decline in exploratory activity in the Pilo-LAT group detected by a decrease in time spent exploring holes (Figure 9A) and rearing (Figure 9B). Anakinra treatment restored the exploratory behavior to typical values. In contrast, in the chronic phase of the model, we observed an increase in climbing, another parameter of exploratory activity (Figure 9C). The time spent exploring holes and rearing was not affected in epileptic animals. Anakinra therapy did not affect either parameter.

#### 2.5.3. Cognitive Functions and Memory

**Figure 10:**
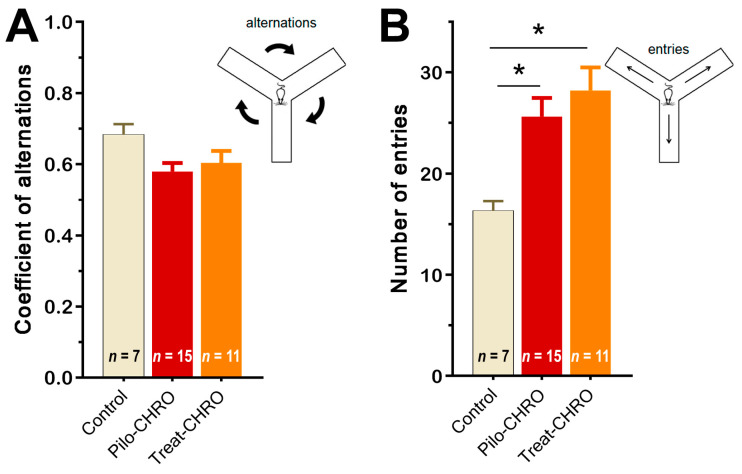
Anakinra treatment did not change the behavior of post-SE rats in the Y maze. One-way ANOVA with Tukey’s post hoc test was used for statistical analysis. Diagrams show the average coefficient of (**A**) alternations and (**B**) the number of visited arms in the Y maze in different groups of rats. Pilo-CHRO: rats with pilocarpine-induced SE studied during the chronic phase of the model accordingly; Treat-CHRO: post-SE rats treated with anakinra. * *p* < 0.05 in ANOVA Tukey’s post hoc test.

Cognitive functions and memory were investigated in the chronic phase of the model using the Y maze test and fear conditioning paradigm. In the Y maze, we did not observe any changes in the coefficient of alternation, suggesting that spatial working memory was not affected by SE or anakinra therapy (Figure 10A). We did observe an increase in the number of visited arms in epileptic animals (F_(2,27)_ = 6.8, *p* < 0.01), which supports the findings from the OFT of an increase in motor activity in these rats. Anakinra treatment did not change the number of arms visited (Figure 10B).

**Figure 11:**
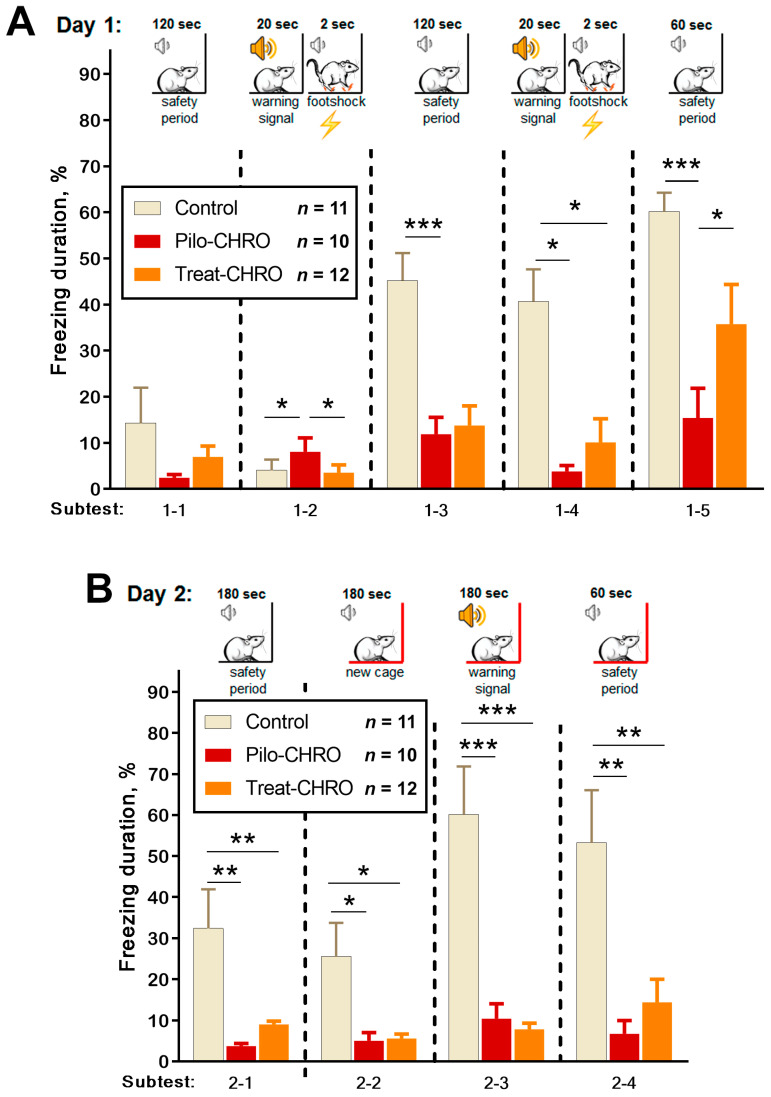
Fear conditioning testing. One-way repeated measures ANOVA with Tukey’s post hoc test was used for statistical analysis. Diagrams show the relative duration of freezing of control and post-SE rats in different subtests during (**A**) training day subtests: 1–2 (F_(2,36)_ = 9.0, *p* < 0.001), 1–3 (F_(2,34)_ = 9.4, *p* < 0.001), 1–4 (F_(2,33)_ = 6.2, *p* < 0.01), 1–5 (F_(2,37)_ = 13.8, *p* < 0.001); and (**B**) testing day in Cage A (subtest 2–1 F_(2,32)_ = 9.4, *p* < 0.001) and new Cage B subtests: 2–2 (F_(2,35)_ = 7.7, *p* < 0.01), 2–3 (F_(2,34)_ = 25, *p* < 0.001) and 2–4 (F_(2,35)_ = 8.9, *p* < 0.001). * *p* < 0.05, ** *p* < 0.01, *** *p* < 0.001: the difference between groups according to the Tukey’s post hoc test.

Fear memory was assessed using the fear conditioning test based on the time of freezing in response to a pain-associated stimulus. On Day 1, we observed a disturbance in fear reaction in the Pilo-CHRO group; the duration of freezing after foot shocks (Subtests 1–3 and 1–5) was smaller than in the control group (Figure 11A). In the group treated with anakinra, the duration of freezing was between that of the control and epileptic groups. These results indicate that anakinra therapy had a positive effect on short-term memory in the fear conditioning paradigm.

On Day 2, we examined context and conditional memory (Figure 11B). In contrast to the control rats, animals from the Pilo-CHRO and Treat-CHRO groups did not demonstrate a fear reaction after placement in Cage A, in which they had received a foot shock the day before. In the new Cage B, a conditional sound did not induce a freezing reaction in epileptic animals, while control animals exhibited prolonged freezing. These results suggest that both types of memories are significantly impaired, and anakinra therapy was not beneficial for the correction of long-term memory associated with aversive stimulation because the freezing duration was considerably shorter than in the control animals (Figure 11B).

#### 2.5.4. Social Interaction

**Figure 12:**
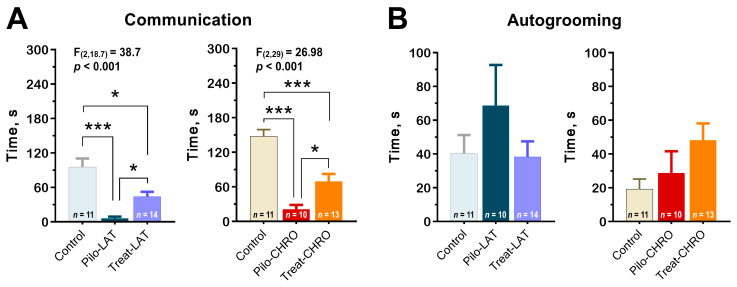
Anakinra treatment attenuates impairments in the social behavior in post-SE rats. One-way ANOVA with Tukey’s or Games–Howell’s post hoc tests was used for statistical analysis. (**A**) Diagram shows the average time of rat communication in the social interaction test (latent phase of the model: F_(2,30)_ = 12.7, *p* < 0.001, and chronic phase: F_(2,29)_ = 27.0, *p* < 0.001). (**B**) Total time of autogrooming is similar in different groups of rats. * *p* < 0.05, ** *p* < 0.01, *** *p* < 0.001.

Using the paradigm of social interaction test, we found a decrease in time spent in active social interaction among post-SE rats in the latent and chronic phases. Anakinra therapy significantly improved communicative behavior during both stages of the model (Figure 12A). However, the duration of communicative acts did not get control values.

Using this test, we did not find differences in anxiety levels measured by the total autogrooming time (Figure 12B).

#### 2.5.5. Sucrose Preference Test

**Figure 13:**
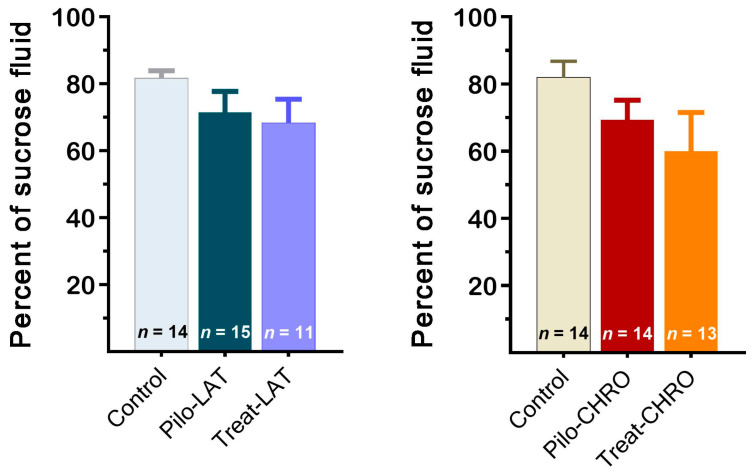
The percentage of sucrose solution consumption was similar in different groups of rats. Pilo-LAT and Pilo-CHRO: rats with pilocarpine-induced SE studied during the latent phase and chronic phase of the model accordingly; Treat-LAT and Treat-CHRO: post-SE rats treated with anakinra and studied during the latent phase and chronic phase of the model.

The sucrose preference test did not reveal anhedonia behavior in any of the tested groups. The sucrose preference was not significantly different in post-SE rats compared with the control group (Figure 13). Thus, our results suggest that neither pilocarpine-induced SE nor anakinra treatment led to depressive-like behavior.

## 3. Discussion

Our results demonstrate that anakinra treatment following SE significantly suppressed the duration and frequency of SRS at the chronic phase of the lithium–pilocarpine model. It also had a neuroprotective effect. However, the treatment did not decrease the mortality rate in the treated group; furthermore, neuroinflammation and gliosis were not significantly suppressed. Results of the behavioral tests showed that anakinra treatment prevented disturbances in locomotor, exploratory, and social activity during the latent phase of the model. In the chronic phase, the therapy demonstrated positive long-term effects on social behavior and fear reaction; however, it did not improve locomotor and exploratory activity or long-term memory.

### 3.1. Methodological Considerations

Anakinra is a human recombinant protein with a small molecular weight. This drug currently has widespread clinical usage because it influences the primary mechanism of various chronic inflammatory diseases. It is thought to have outstanding therapeutic potential due to its natural origin, high binding affinity and target specificity, and low toxicity. However, the efficiency of anakinra may be limited by two characteristics: low brain uptake and short biological half-life (4–6 h) [25,26].

In clinical practice, anakinra is usually used in the treatment of autoinflammatory disorders, but there are several reports about anakinra usage in refractory epilepsy [27]. In a child with febrile infection-related epilepsy syndrome, anakinra therapy decreased the frequency of seizures, and it returned proinflammatory cytokines in cerebrospinal fluid to a normal level [28]. Anakinra treatment led to the reduction or even termination of intractable chronic seizures throughout the therapy in patients in another study [29]. The anticonvulsant effect of anakinra has been shown in animal models of acute SE [18,30,31,32]. However, the antiepileptogenic activity and disease-modifying effects of anakinra treatment are still unclear.

In this study, we used high doses of anakinra in i.p. injections (50–100 mg/kg) because it has been shown that a therapeutic concentration in the brain is low and can only be achieved by peripheral injection of a high dose [33,34]. Another possible limitation of our study is that anakinra was administered only one time per day. Therefore, drug action was most likely pulsatory, and the effect might be different if other schedules of administration were used.

### 3.2. The Potential Mechanisms of the Antiepileptogenic Effect of Anakinra Treatment: Gene Expression and Histological Findings

In this study, we hypothesized that the administration of anakinra would attenuate the IL-1β-mediated proinflammatory pathway, consequently reducing neuroinflammation and preventing epileptogenesis. However, our hypothesis was only partially confirmed. We found that anakinra administration did not affect IL-1β and TNFα gene expression in the latent phase in the DH, VH, or TC, which are the structures most significantly involved in epileptogenesis in the lithium–pilocarpine model. We suggest that the main effects of the treatment are mostly due to changes in the IL-1β/IL-1Ra ratio but not due to a direct reduction in IL-1β or other proinflammatory cytokine levels.

The neuroprotective effect of anakinra treatment might be explained by its impact on properties of glutamatergic neurotransmission. In neurons, Il-1R1s are broadly colocalized with NMDA receptors [35], especially its GluN2B subunit [36]. The activation of Il-1R/TLR4 signaling in neurons leads to ceramide/src kinase-mediated phosphorylation of the GluN2B-containing NMDA receptors [37]. A 50% increase in NMDA current was found after the Il-1β application to hippocampal neurons culture [38]. Increased calcium influx through NMDA receptors results in excitotoxicity and cell loss [39]. Therefore, the presence of anakinra may prevent overactivation of GluN2B-containing NMDA receptors and excitotoxicity.

Another critical finding that might be relevant to the antiepileptogenic effect of anakinra treatment is the hyperexpression of the *Slc1a2* gene in the VH. The *Slc1a2* gene encodes EAAT2, an astrocytic glutamate transporter, which provides ~90% of glutamate reuptake from the synaptic cleft [40]. Extracellular glutamate plays a crucial role in the initiation of seizures [41]. The hyperexpression of EAAT2 has prevented or weakened seizures in different models [42,43,44,45] and had a neuroprotective effect [46]. Indeed, we found that anakinra treatment partially precluded neuronal loss in the hippocampus. It should be noted, however, that in the TC, *Slc1a2* gene expression was slightly decreased following anakinra treatment.

However, anakinra treatment did not prevent gliosis. We observed an increase in the expression of *Gfap* and *Itpr2* genes following pilocarpine-induced SE in both treated and nontreated groups. In line with molecular data, we observed a significant increase in the number of astrocytes and microglial cells.

### 3.3. Behavioral Data

Inflammatory processes have been found to be associated with various comorbidities of epilepsy, such as cognitive dysfunction [47,48], depression [49,50], and psychosis [51]. It has been suggested that neuroinflammation could be the main link between epilepsy and its psychiatric comorbidities [52,53]. Thus, anti-inflammatory therapy can be beneficial in terms of preventing epilepsy-associated cognitive and emotional disturbances.

We previously described behavioral disturbances in the lithium–pilocarpine model in detail [24]. The main disturbances are motor hyperactivity, decreased social communication, and altered memory functions. Based on these results, we chose a set of behavioral tests mainly associated with impaired forms of behavior: an OFT for motor and explorative activity, a social interaction test for communication, Y maze and fear conditioning tests for different forms of memory, and a sucrose preference test for depressive-like behavior.

During anakinra treatment, post-SE rats did not exhibit hyperactivity. Their social behavior was similar to that of control rats. Moreover, we did not observe any adverse side effects of treatment. The long-term effects of anakinra treatment on rats’ behavior were less profound. Motor hyperactivity and long-term memory function were not amended by therapy in epileptic rats. However, the alterations in working memory function and social interaction observed in the chronic phase of the model were partially improved by anakinra administration.

## 4. Materials and Methods

### 4.1. Animals

Wistar rats were bred at the animal facility of the Sechenov Institute of Evolutionary Physiology and Biochemistry of the Russian Academy of Sciences (Saint Petersburg, Russia). The rats were housed in standard home cages (six to seven rats per cage) with free access to food and water and a 12 h dark–light cycle (8 p.m. to 8 a.m.). Rats in control and experimental groups were mixed from different litters to avoid any influence of genetic factors. All the experiments were carried out under the Guidelines on the Treatment of Laboratory Animals effective at the Sechenov Institute of Evolutionary Physiology and Biochemistry of the Russian Academy of Sciences (Ethical Permit Number: 13-k-a, 15 February 2018). These guidelines comply with EU Directive 2010/63/EU for animal experiments.

### 4.2. Lithium–Pilocarpine Model and Treatment

Seven-week-old male rats were injected intraperitoneally (i.p.) with 127 mg/kg of lithium chloride (LiCl; Sigma-Aldrich, St. Louis, MO, USA) 1 day before pilocarpine injection. At 1 h before pilocarpine injection, (−)-scopolamine methyl bromide (1 mg/kg, i.p.; Sigma-Aldrich) was administered to block the peripheral muscarinic receptors. Rats received one or several injections of pilocarpine (i.p.; Sigma-Aldrich), depending on the intensity of induced seizures. Seizure severity was estimated according to the Racine scale: (1) facial automatism, (2) head-nodding, (3) forelimb myoclonus, (4) rearing, (5) rearing and falling, (6) wild running, and (7) generalized clonic-tonic convulsions [54]. The first dose of pilocarpine was 10 mg/kg. If the rat did not exhibit seizures of score 4 or above within 30 min, additional doses of 10 mg/kg of pilocarpine were injected every 30 min. Rats that did not produce convulsions of score 4 or above after the fourth injection (total dose of 40 mg/kg) were excluded from further experiments. Diazepam (10 mg/kg, i.p.; Sigma-Aldrich) was administered 75 min after the beginning of seizures of score 4 to cease convulsions. Therefore, all rats included in the study developed SE. The total number of animals used in the study was 153.

**Figure 14:**
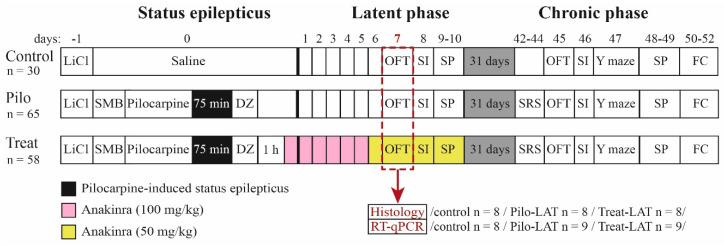
Experimental design. Pilo: rats administered with pilocarpine, Treat: rats administered with pilocarpine and then treated by anakinra, LiCl: lithium chloride, SMB: (−)-scopolamine methyl bromide, DZ: diazepam, OFT: open-field test, SI: social interaction test, SP: sucrose preference test, Y maze: Y-shaped maze spontaneous alternation test, FC: fear conditioning, SRS: spontaneous recurrent seizure registration, RT-qPCR: reverse transcription followed by a quantitative polymerase chain reaction.

The design of the study is shown in Figure 14. The experiments were performed during the latent and chronic phases of the model using three groups of rats: (1) control (administered with saline instead of pilocarpine), (2) Pilo (rats administered with pilocarpine that developed SE), and (3) Treat (post-SE anakinra-treated rats).

Anakinra was obtained using the *Escherichia coli* TG1 (pTAC-hIL-1ra)-producing strain (Institute of Highly Pure Biopreparations, St. Petersburg, Russia). The protein (99% purity) was prepared at a concentration of 100 mg/mL in 0.02 M phosphate buffer pH 7.0 0.14 M NaCl; 0.01% polysorbate 80. The protein has been shown to block the IL-1β-stimulated proliferation of mouse T-lymphocytes by 50% at doses less than 10-times greater than the IL-1β dose.

Anakinra (100 mg/kg) was administered 1 h after diazepam injection and then daily for the next 5 days. For Days 6–10, anakinra was administered in a dose of 50 mg/kg. The dosage was determined based on previous experimental works [25,55,56,57].

### 4.3. Survival and Body Weight

Survival and body weight were monitored for 2 weeks after SE. The first week after SE, rats were administered a 5% glucose solution (2 mL, subcutaneous) daily to improve survival [58].

### 4.4. Spontaneous Recurrent Seizures

The presence of SRS was evaluated 6 weeks after SE. Each rat was placed in a transparent cage with water and food for 16 h (9 p.m. to 1 p.m.) for 3 consecutive days and videotaped. The duration of each SRS episode was determined. Then, we calculated the total and average time and frequency of SRS.

### 4.5. Behavioral Testing

Behavioral testing was performed in the latent (from Days 7 to 10 following pilocarpine-induced SE) and chronic (from Days 45 to 52) phases of the lithium–pilocarpine model (Figure 14).

#### 4.5.1. Open-Field Test

An open-field test (OFT) [59] was used to assess motor and explorative activity. The open-field arena had a diameter of 1 m, wall height of 30 cm, illumination of 8 Lx, and 4 cm round holes in the floor. The rat was placed in the center of the arena. Each rat’s movement was recorded for 3 min. The recordings were analyzed offline using the Round and Cross and Field4W software (Institute of Experimental Medicine, St. Petersburg, Russia). We defined the form of the tracks and locomotor characteristics in the different field zones. The time spent in the center of the arena (1/4 of arena radius) and near the wall (less than 20 cm from the wall, thigmotaxis) were measured to estimate anxiety level. Total distance, average speed, time of locomotion, and immobility were calculated to determine locomotor activity. The number and total duration of the following behavioral patterns were measured: hole exploration, sniffing, climbing, and rearing (explorative activity) as well as locomotion and actions in a place (locomotor activity).

#### 4.5.2. Y-Shaped Maze Spontaneous Alternation Test

The Y maze [60] was used to measure spatial working memory. The maze consisted of three arms (each 50 × 10 cm) with opaque, 30 cm high walls. The rat was placed in the center, and the sequence of entries into arms was analyzed for 8 min. Entry was considered correct if it differed from two previous entries, for instance, 1-2-3, 2-3-1, or 1-3-2. Repeated numbers, such as 1-1, were considered two entries. The coefficient of alternation (C_A_) was used as an index of operative spatial memory. It was calculated as follows: C_A_ = N_right_/(N_total_ − 2), where N_right_ is the number of correct entries into a new arm, and N_total_ is the total number of entries.

#### 4.5.3. Fear Conditioning Test

The fear conditioning test of short-term and long-term fear-associated memory [61] was performed over 3 days. Two Plexiglas cages were used in this test. Cage A (45 × 30 cm, height = 20 cm) had an electroconductive floor. Cage B was larger (60 × 30 cm, height = 40 cm) and had no electroconductive floor. On Day 0 (habituation day), the rat habituated to the conditioning of Cage A for 3 min. On Day 1 (conditioning day), the rat was placed into Cage A. Five steps of conditioning were performed: 120 s of habituation (Step 1-1), 20 s of an auditory cue (80 dB sound) followed by 2 s of mild (0.6 mA) foot shock through the electroconductive floor (Step 1-2), a 120 s break (Step 1-3), repetition of Step 1-2 (Step 1-4), and 60 s of rest (Step 1-5). On day 2 (testing day), rats were placed in Cage A for 3 min (Step 2-1) without a cue or current to estimate condition-associated fear (contextual conditioning testing). The rat was then moved to Cage B (Steps 2-2 to 2-4), which was a different size and had pictures of geometrical figures on the walls and a vanillin drop on the floor to create a new odor that made Cage B unfamiliar to the rat. Thus, Cage B was not associated with Cage A. After 3 min of habituation (Step 2-2), the same current-associated sound was given for 3 min (Step 2-3) to estimate cue-associated fear (cued conditional testing). No stimuli were given during the last 1 min step (Step 2-4).

During each step, the total time of freezing was measured to estimate fear response to an aversive stimulus. Since the length of steps was different, the relative time of freezing (% of step duration) was used to measure fear-associated memory. Freezing during Steps 1-3 and 1-5 (immediately after the foot shock) reflected short-term fear-related memory.

#### 4.5.4. Social Interaction Test

A social interaction test was used to estimate social behavior [62]. Rats were placed into a Plexiglas cage (60 × 30 cm, height = 40 cm) for 30 min before the test to decrease anxiety from the new environment [62]. Then, an unfamiliar, adult, intact male Wistar rat was placed into the same cage for 5 min. The following patterns were measured: communication (sniffing and grooming the intruder’s body and sniffing intruder’s tail and genitalia), aggression, defense, sexual-like behavior to a male intruder (mounting and genitalia licking after mounting), and noncommunicative behavior (autogrooming).

#### 4.5.5. Sucrose Preference Test

The sucrose preference test [63] was performed for 2 consecutive days to estimate depressive-like behavior (anhedonia). On Day 1, a bottle with a 1% sucrose solution was placed in the home cage so that the rats could grow accustomed to the sweet taste. On Day 2, the rats were put into individual cages (30 × 30 cm, height = 40 cm, one rat per cage) with two bottles, the first one containing plain drinking water and the other containing 1% sucrose solution. After 18 h (6 p.m. to 12 p.m.), the water and sucrose solution intakes were measured by weighing. The sucrose preference was calculated as the ratio of sucrose solution consumption to total liquid consumption.

### 4.6. Histology

Seven days after pilocarpine-induced SE, rats were anesthetized with chloral hydrate (400 mg/kg, i.p.). Following transcardial perfusion with a 4% solution of paraformaldehyde (PFA) in 0.1 M phosphate-buffered saline (PBS, pH 7.4), brains were fixed in PFA for 2 weeks, then frozen and sectioned (15 μm thickness) at the coronal plane using cryomicrotome Leica CM 1510S (Leica Microsystems, Wetzlar, Germany). The analyzed blocks of dorsal hippocampus (DH) tissue began at 3.3 mm from the Bregma [64], from six to ten sections per brain used for each type of analysis. For random sampling, the first of the sections in the analyzed sequence was chosen randomly, and the distance between analyzed sections was 60 µm.

Analysis of neuronal loss in the hippocampal areas CA1 and CA3 was carried out using a cresyl violet (Nissl)-stained section under an AF7000 microscope (Leica Microsystems, Wetzlar, Germany). The image was digitized using a DFC495 camera (Leica Microsystems).

The distribution of the neuronal and glial markers was analyzed using indirect immunofluorescence analysis. Sections were incubated overnight at 37 °C in PBS containing 2% bovine serum albumin, 0.3% Triton X-100 (Merck, Darmstadt, Germany), and one of three primary antibodies developed in rabbits: neuronal marker anti-NeuN (Abcam, Cambridge, UK, ab104225, 1:200 dilution), astrocyte marker anti-GFAP (Abcam, ab7260, 1:200), or microglial marker anti-Iba1 (Abcam, ab178846, 1:100). After thorough rinsing, the sections were incubated in fluorescent-tagged secondary antibodies for 1 h at 37 °C. Anti-NeuN and anti-GFAP were visualized with phycoerythrin-conjugated (FITC) secondary antibodies to rabbit IgG (Abcam, ab97050, 1:200), and anti-Iba1 was visualized with FITC secondary antibodies to rabbit IgG (Abcam, ab7007, 1:200). Immunofluorescence studies were performed on a Leica DMR microscope connected to the Leica TCS SL confocal scanner (Leica Microsystems). Fluorochromes were excited by a He/Ar laser at a wavelength of 488 nm. The FITC fluorescence signal was observed in the wavelength range of 496–537 nm and the phycoerythrin signal in the wavelength of 652–690 nm. The Video Test Master-Morphology program (Video Test, Moscow, Russia) was used to count the number of immunopositive cells. A rectangular, 500 μm window was applied at specific landmarks in the CA1 or CA3 areas. The cell was considered immunopositive if it differed in brightness from the background at three or more points.

### 4.7. mRNA Expression Analysis

Rats were decapitated 7 days after SE. The brain was quickly extracted and frozen at −80 °C. DH and ventral hippocampus (VH) areas and the temporal cortex (TC) were dissected using a micro spatula and stored at −20 °C in the OTF5000 Cryostat Microtome (Bright Instruments, Luton, UK) according to the rat brain atlas [65]. Total RNA was extracted by a single-step acid guanidinium thiocyanate–phenol–chloroform method [66] using the ExtractRNA reagent (Evrogen, Moscow, Russia) according to the manufacturer’s instructions. RNA samples were treated with 1 unit of RQ1 DNAse (Promega, Madison, WI, USA) for 15 min followed by LiCl precipitation and ethanol washing. RNA concentration and purity were assessed spectrophotometrically based on a 260 nm absorbance and a 260/280 absorbance ratio, respectively, using the NanoDrop™ Lite Spectrophotometer (Thermo Fisher Scientific, Waltham, MA, USA).

cDNA was synthesized from 1 μg (VH) or 2 μg (TC and DH) of total RNA, with oligo-dT (0.5 µg per 1 µg of RNA) and 9-mer random (0.25 µg per 1 µg of RNA) primers (DNA Synthesis Ltd., Moscow, Russia) and M-MLV reverse transcriptase (100 units per 1 µg of RNA; Promega, Madison, WI, USA) in a total volume of 25 µL following the manufacturer’s instruction. Briefly, 8 µL of an RNA solution was mixed with primers and incubated for 10 min at 70 °C. This was quickly cooled to 4 °C for primer annealing, then a mixed revertase containing a reaction mix was added, and samples were incubated for 1 h at 42 °C and 10 min at 65 °C. All samples were diluted 10-fold before the PCR step. qPCR was performed in a total volume of 10 µL with 0.8 µL of cDNA, 0.75 units of TaqM-polymerase (Alkor Bio, St. Petersburg, Russia), and 3.5 mM of Mg^2+^; specific forward primers, reverse primers, and hydrolysis (TaqMan) probes were inserted at assay-dependent concentrations (see Appendix A Table A1). Nucleotides were purchased from DNA Synthesis Ltd. (Moscow, Russia). qPCR for inositol 1,4,5-trisphosphate receptor type 2 (*Itpr2*) was performed as singleplex. Other reactions were multiplexed as follows: *Il1b* with *Tnfa*, glial fibrillary acidic protein (*Gfap*) with solute carrier family 1 member 2 gene (*Slc1a2,* encoding excitatory amino acid transporter 2 (EAAT2), and three triplex qPCR assays for reference genes *Actb + Gapdh + B2m*, *Rpl13a + Ppia + Sdha*, and *Hprt1 + Pgk1 + Ywhaz* as previously described [67]. PCR efficiencies were assessed using the serial dilution method [68]. All assays demonstrated optimal efficiencies within the 90–105% range; multiplexed reactions demonstrated performance compatible with singleplex. PCR reactions were triplicated and carried out in a C1000 Touch thermal cycler combined with a CFX96 Touch™ Real-Time PCR Detection System (BioRad, Hercules, CA, USA) simultaneously with no template and no reverse transcription control samples. Reference genes for normalization of expression data were selected based on comprehensive ranking obtained using the RefFinder online tool (https://www.heartcure.com.au/reffinder/) incorporated with the GeNorm [69], NormFinder [70], BestKeeper [71], and comparative deltaCT [72] algorithms.

The relative expressions of the Gfap, *Slc1a2*, *Itpr2*, *Il1b*, and *Tnfa* genes were calculated using the 2−ΔΔCt method normalized against the geometric average for the three-most stable analyzed brain region reference genes [73]: *Ppia*, *Ywhaz*, and *Pgk1* in the DH and VH and *Gapdh*, *Pgk1*, and *Rpl13a* in the TC.

### 4.8. Statistical Analysis

Statistical analysis was performed with SPSS Statistics 23 (IBM, Armonk, New York, NY, USA) and StatSoft Statistica 8 (TIBCO, Palo Alto, CA, USA). For the survival analysis, we used the Kaplan–Meier procedure with Breslow’s criterion for the test of distribution equality.

Iglewicz and Hoaglin’s robust test for multiple outliers was used for the identification and rejection of outliers. The Kolmogorov–Smirnov and Shapiro–Wilk tests were used to examine the normality of distribution. Student’s *t*-test and one-way and repeated measures ANOVA with Tukey’s post hoc test were used for normally distributed data. Welch’s ANOVA and the Games–Howell post hoc test were used if the assumption of homogeneity of variances was violated. The nonparametric Kruskal–Wallis test followed by Dunn’s post hoc test were used to compare the number of immunopositive cells in different experimental groups. For all tests, group differences were considered statistically significant at the *p* < 0.05 level. All histograms represent means ± SEMs unless otherwise specified.

## 5. Conclusions

This study demonstrated that anakinra is a promising drug candidate as a preventive treatment for epileptogenesis. Although anakinra showed imperfect efficiency in the lithium–pilocarpine model, it did not have any apparent adverse side effects. Whether a combination of anakinra with another compatible cure may increase its therapeutic efficiency requires additional research. This study is a step toward finding a treatment for the prevention of epilepsy in patients who are at risk of developing seizures [6].

## Appendix A

**Table A1:** Primers and probes used in qRT-PCR.

Gene Symbol	Nucleotide Sequences	Reference
RefSeq Accession Number	(Forward, Reverse, TaqMan-Probe)	
*Il1b*	CACCTCTCAAGCAGAGCACAG	[74]
	GGGTTCCATGGTGAAGTCAAC	
NM_031512	TGTCCCGACCATTGCTGTTTCCTAG ^#^	
*Tnfa*	CCAGGTTCTCTTCAAGGGACAA	[74]
NM_012675	CTCCTGGTATGAAATGGCAAATC	
	CCCGACTATGTGCTCCTCACCCACA *	
*Gfap*	TGGCCACCAGTAACATGCAA	[75] (forward and reverse primers)
NM_017009.2	CAGTTGGCGGCGATAGTCAT	This work (hydrolysis probe)
	CGGTCCAAGTTTGCAGACCTCACAG ^&^	
*Slc1a2*	CCAGTGCTGGAACTTTGCCT	[76] (forward and reverse primers)
NM_001035233.1	TAAAGGGCTGTACCATCCAT	This work (hydrolysis probe)
	AGCGTGTGACCAGATTCGTCCTCCCA ^#^	
*Itpr2*	CTACAACGACCGAGCCTCAT	[77] (forward and reverse primers)
NM_031046.3	CCACCCTCACTATGTCGTCC	This work (hydrolysis probe)
	AGCCCGTGGGGATGAGAGCGGCCC *	
*Gapdh*	TGCACCACCAACTGCTTAG	[78]
NM_017008	GGATGCAGGGATGATGTTC	
	ATCACGCCACAGCTTTCCAGAGGG ^&^	
*Ppia*	AGGATTCATGTGCCAGGGTG	[79]
NM_017101	CTCAGTCTTGGCAGTGCAGA	
	CACGCCATAATGGCACTGGTGGCA *	
*B2m*	TGCCATTCAGAAAACTCCCC	[80]
NM_012512	GAGGAAGTTGGGCTTCCCATT	
	ATTCAAGTGTACTCTCGCCATCCACCG *	
*Actb*	TGTCACCAACTGGGACGATA	[81] (forward and reverse primers)
NM_031144	GGGGTGTTGAAGGTCTCAAA	[67] (hydrolysis probe)
	CGTGTGGCCCCTGAGGAGCAC ^#^	
*Ywhaz*	GATGAAGCCATTGCTGAACTTG	[82] (forward and reverse primers)
NM_013011	GTCTCCTTGGGTATCCGATGTC	[67] (hydrolysis probe)
	TGAAGAGTCGTACAAAGACAGCACGC *	
*Rpl13a*	GGATCCCTCCACCCTATGACA	[83] (forward and reverse primers)
NM_173340	CTGGTACTTCCACCCGACCTC	[67] (hydrolysis probe)
	CTGCCCTCAAGGTTGTGCGGCT ^#^	
*Pgk1*	ATGCAAAGACTGGCCAAGCTAC	[82] (forward and reverse primers)
NM_053291	AGCCACAGCCTCAGCATATTTC	[67] (hydrolysis probe)
	TGCTGGCTGGATGGGCTTGGA ^&^	
*Hprt1*	TCCTCAGACCGCTTTTCCCGC	[84] (forward and reverse primers)
NM_012583	TCATCATCACTAATCACGACGCTGG	[67] ] (hydrolysis probe)
	CCGACCGGTTCTGTCATGTCGACCCT ^#^	
*Sdha*	AGACGTTTGACAGGGGAATG	[85] (forward and reverse primers)
NM_130428	TCATCAATCCGCACCTTGTA	[67] (hydrolysis probe)
	ACCTGGTGGAGACGCTGGAGCT ^&^	

^&^ Labeled with R6G fluorescent dye and BHQ2 quencher; ^#^ Labeled with FAM fluorescent dye and BHQ1 quencher; * Labeled with ROX fluorescent dye and BHQ1 quencher.
